# Roles of OA1 octopamine receptor and Dop1 dopamine receptor in mediating appetitive and aversive reinforcement revealed by RNAi studies

**DOI:** 10.1038/srep29696

**Published:** 2016-07-14

**Authors:** Hiroko Awata, Ryo Wakuda, Yoshiyasu Ishimaru, Yuji Matsuoka, Kanta Terao, Satomi Katata, Yukihisa Matsumoto, Yoshitaka Hamanaka, Sumihare Noji, Taro Mito, Makoto Mizunami

**Affiliations:** 1 Faculty of Science, Hokkaido University, Sapporo, 060-0810, Japan; 2 Graduate School of Live Sciences, Hokkaido University, Sapporo, 060-0810, Japan; 3 Department of Life Systems, Institute of Technology and Science, Tokushima University, Tokushima 770-8506, Japan; 4 Faculty of Liberal Arts, Tokyo Medical and Dental University, Ichikawa 272-0827, Japan

## Abstract

Revealing reinforcing mechanisms in associative learning is important for elucidation of brain mechanisms of behavior. In mammals, dopamine neurons are thought to mediate both appetitive and aversive reinforcement signals. Studies using transgenic fruit-flies suggested that dopamine neurons mediate both appetitive and aversive reinforcements, through the Dop1 dopamine receptor, but our studies using octopamine and dopamine receptor antagonists and using Dop1 knockout crickets suggested that octopamine neurons mediate appetitive reinforcement and dopamine neurons mediate aversive reinforcement in associative learning in crickets. To fully resolve this issue, we examined the effects of silencing of expression of genes that code the OA1 octopamine receptor and Dop1 and Dop2 dopamine receptors by RNAi in crickets. *OA1*-silenced crickets exhibited impairment in appetitive learning with water but not in aversive learning with sodium chloride solution, while *Dop1*-silenced crickets exhibited impairment in aversive learning but not in appetitive learning. *Dop2*-silenced crickets showed normal scores in both appetitive learning and aversive learning. The results indicate that octopamine neurons mediate appetitive reinforcement via OA1 and that dopamine neurons mediate aversive reinforcement via Dop1 in crickets, providing decisive evidence that neurotransmitters and receptors that mediate appetitive reinforcement indeed differ among different species of insects.

Much effort has been made to elucidate reinforcing mechanisms for associative learning. In mammals, dopamine neurons in the ventral tegmental area of the midbrain are thought to play critical roles in mediating both appetitive and aversive reinforcement^1^,^2^. In crickets, we observed that administration of octopamine receptor antagonists (epinastine and mianserin) impairs appetitive learning but not aversive learning, whereas administration of dopamine receptor antagonists (flupentixol, fluphenazine, spiperone and chlorpromazine) impairs aversive learning but not appetitive learning, and we thus suggested that octopamine and dopamine neurons mediate appetitive and aversive reinforcement, respectively^3^,^4^,^5^,^6^,^7^,^8^. Pharmacological studies in honey bees also suggested participation of octopamine and dopamine in appetitive and aversive learning, respectively^9^,^10^,^11^.

Bases on results of studies using transgenic fruit-flies, on the other hand, it is thought that different types of dopamine neurons mediate appetitive reinforcement and aversive reinforcement, via Dop1 (also referred to as DUMB or DopR1) dopamine receptor, and that octopamine neurons transmit a reward signal to dopaminergic reinforcing neurons, in part via OA1 (also referred to as OAMB) octopamine receptor^12^,^13^,^14^,^15^,^16^ (for a different view, see in ref. 17). This urged us to reexamine the validity of our suggestion, partly because specificities of receptor antagonists used in our studies are not perfect. We produced *Dop1* knockout crickets using the CRISPR/Cas9 system^18^ and found that *Dop1* knockout crickets exhibit impairment in aversive learning but not appetitive learning^19^. This observation, however, is not decisive, since the possibility that the observed defects are due to defects in brain development cannot be ruled out. One solution to this problem is the use of RNA interference (RNAi): methods to reduce target gene expression by dsRNA injection into the hemolymph have been well established in crickets^20^,^21^,^22^.

In this study we investigated the effects of silencing of the expression of genes that code OA1 octopamine receptor^23^ and Dop1 and Dop2 dopamine receptors^24^ by RNAi on appetitive and aversive olfactory learning in crickets. We selected these three receptors for analysis, among several types of octopamine and dopamine receptors reported in insects^25^,^26^,^27^, because genes for these receptors are known to be expressed in the Kenyon cells of the mushroom body, which play critical roles in olfactory learning^28^,^29^,^30^. For examples, studies with *in situ* hybridization in crickets have shown high levels of expression of *Dop1* and *Dop2* mRNA in Kenyon cells^2^, and a high level of *OA1* mRNA expression is found in Kenyon cells in fruit-flies^31^ and honey bees^32^.

We used olfactory conditioning of the maxillary palpi extension response (MER)^8^ for evaluation of the effects of gene silencing by RNAi. Crickets extend their maxillary palpi and vigorously swing them when water is applied to the antennae or when filter paper soaked with odor essence is presented near their antennae^8^,^33^. We have reported that crickets subjected to pairing of an odor with water as a reward exhibited an increase in the probability of MER to the odor, whereas crickets subjected to pairing of an odor with 20% sodium chloride solution as a punishment exhibited a decrease of MER to the odor^8^.

## Results

### Suppression of gene expression by dsRNA injections of *OA1*, *Dop1* and *Dop2* in the cricket

**Figure 1:**
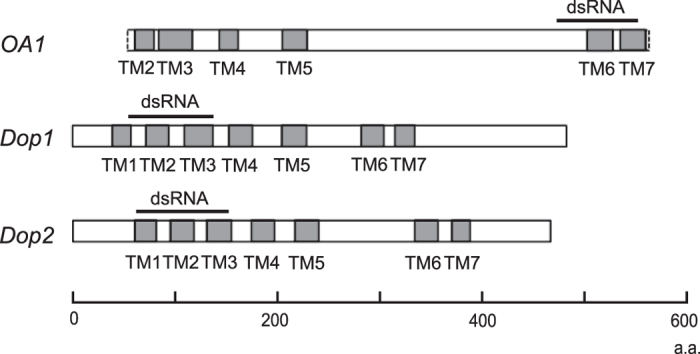
Structures of *OA1*, *Dop1* and *Dop2* genes of the cricket *Gryllus bimaculatus*. The regions of synthesized dsRNA are indicated by black bars. TM indicates the regions encoding transmembrane domains. a.a., amino acids. DNA sequences of *Gryllus bimaculatus* (*Gb*) *Dop1 and Dop2* genes were reported by Watanabe *et al*.^22^ (*Dop1*: AB720739, *Dop2*: AB720740) and that of *Gb OA1* was determined in this study (Accession No.: LC155976). The similarity of amino acid sequence of *Gb* OA1 to that of OA1 of other insects is 95.3% for *Schistocerca gregaria* OA1(OctαR), 75.8% for *Periplaneta americana* OA1(AAP93817) and 74.5% for *Apis mellifera* OA1. The sequences of probes used in this study were identical to those of the genes described here.

**Figure 2:**
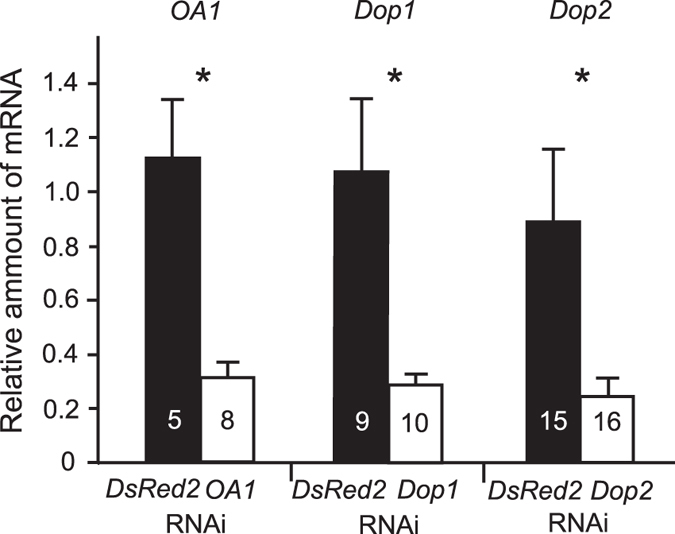
Relative amount of *OA1*, *Dop1* or *Dop2* mRNA in *OA1*, *Dop1* or *Dop2* RNAi crickets in comparison with that in *DsRed2* RNAi crickets revealed by quantitative real-time PCR. Crickets were individually injected with 20 pmol *OA1*, *Dop1*, *Dop2* or *DsRed2* dsRNA. Total RNA was extracted from the heads at 48 hours after injection. cDNA was synthesized using the Superscript First Strand Synthesis System for RT-PCR with random hexamers. The amount of *OA1*, *Dop1* or *Dop2* mRNA in *OA1*, *Dop1* or *Dop2* RNAi crickets relative to that in intact crickets is shown as a white bar. The amount of *OA1*, *Dop1* or *Dop2* mRNA in *dsRed2* RNAi crickets relative to that in intact crickets is shown as a control (black bars). The sample numbers are indicated in the bars. Above the bars, statistical results of a two-sample t-test with Welch’s correction are shown. *p < 0.05.

We first studied the effects of double-stranded RNA (dsRNA) injection of the *OA1, Dop1* or *Dop2* gene on the expression levels of target gene mRNA in the cricket head. As a control, the amount of *OA1, Dop1* or *Dop2* mRNA in the brains of *DsRed2* RNAi crickets was also studied. Groups of crickets were injected with 20 pmol *OA1, Dop1, Dop2* or *DsRed2* dsRNA into the head hemolymph. Target sequences of RNAi for *OA1*, *Dop1* and *Dop2* are shown in Methods and in Fig. 1. The amounts of mRNA in the heads of dsRNA-injected crickets were measured by quantitative real-time PCR (qPCR) at 48 hr after injection, and the relative amount of mRNA transcribed from the target gene was compared to that in intact crickets. The levels of *OA1*, *Dop1* and *Dop2* mRNA in *DsRed2* RNAi crickets were similar to those in intact crickets (Fig. 2). In contrast, expression levels of *OA1, Dop1* and *Dop2* mRNA in the heads of *OA1*, *Dop1* and *Dop2* RNAi crickets were, on average, 31%, 29% and 25% of those in intact crickets. Statistical comparisons showed that the levels of *OA1*, *Dop1* and *Dop2* mRNA in *OA1*, *Dop1* and *Dop2* RNAi crickets were significantly lower than those in *DsRed2* RNAi crickets (*OA1*: t = 3.68, df = 4.60, p = 0.017; *Dop1*: t = 3.09, df = 8.26, p = 0.014; *Dop2*: t = 2.31, df = 15.28, p = 0.035, two sample t-test with Welch’s correction). All RNAi crickets exhibited normal viability, and their locomotor activities and feeding behaviors were not distinguishable from those of intact animals. Evidence suggesting intact sensory and motor functions of those RNAi crickets is described below.

### Effects of the suppression of *OA1, Dop1* and *Dop2* genes on acquisition and retention in appetitive conditioning

We then studied the effects of gene silencing by RNAi on acquisition and retention performances in appetitive olfactory conditioning. We used conditioning of maxillary palpi extension response (MER) for evaluation of the conditioning effect^8^. We have reported that presentation of peppermint or apple odor to the antennae of crickets rarely (in less than 20% of crickets) induces MER and that pairing of each odor with water leads to an increase in the probability of MER^8^.

**Figure 3:**
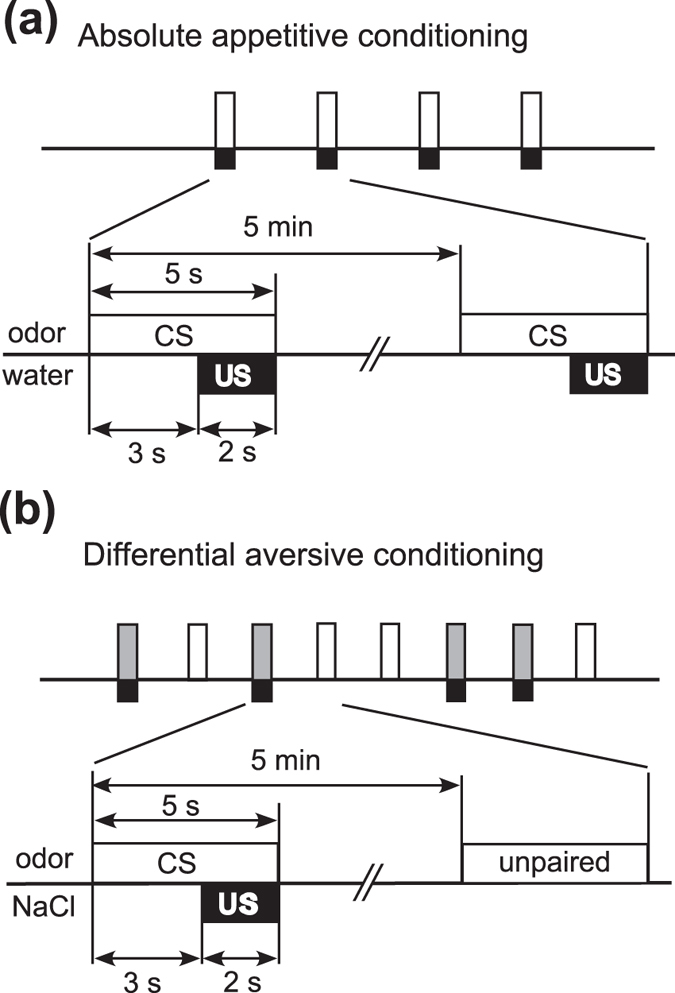
Procedures of appetitive and aversive conditioning. (**a**) Procedures for absolute appetitive conditioning with water reward. Crickets were subjected to four conditioning trials to associate an odor (CS) with water (appetitive US) with an inter-trial interval (ITI) of 5 min. (**b**) Procedures for differential aversive conditioning with 20% sodium chloride solution (punishment). Crickets were subjected to presentation of one odor (paired odor, CS) paired with sodium chloride solution (aversive US) and another odor (unpaired odor) without pairing with US four times each with pseudo-random sequences and with 5-min intervals.

We first studied acquisition performance in appetitive conditioning in groups of crickets injected with dsRNA. Four groups of crickets were injected with 20 pmol dsRNA of *OA1*, *Dop1*, *Dop2* or *DsRed2*. Two days after the injection, they were subjected to 4-trial conditioning to associate an odor with water reward with inter-trial intervals of 5 min. The absence or presence of MER to 3-sec odor (CS) presentation prior to presentation of water (US) was recorded (Fig. 3a).

**Figure 4:**
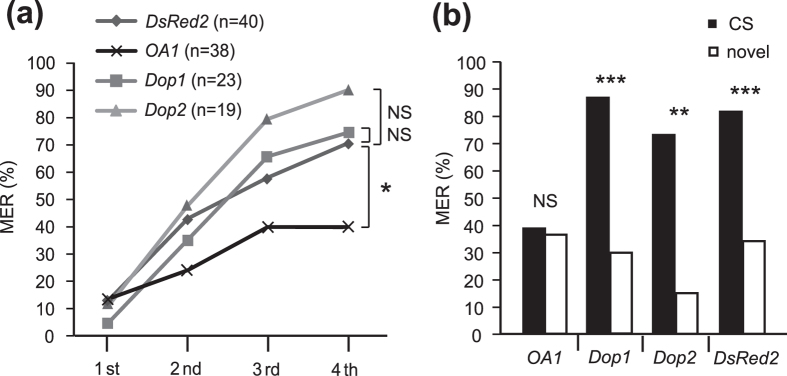
Acquisition and retention in appetitive conditioning of *OA1, Dop1* and *Dop2* RNAi crickets. (**a**) Acquisition performance. Crickets were injected with *OA1*, *Dop1*, *Dop2* or *DsRed2* dsRNA, and two days later they were subjected to 4-trial appetitive conditioning to associate an odor with water with 5-min inter-trial intervals. Percentages of MER to the odors paired with the US (paired odor) are plotted. Results of statistical comparison among groups in the 4th trial are shown (Fisher’s exact test, adjusted by Holm’s method). The sample numbers are shown in parenthesis. (**b**) Retention performance at 30 min after conditioning. Percentages of MER to the paired odor (white bar) and the odor not presented in training (novel odor) (black bar) are shown. Above the bars, statistical results of McNemar’s test are shown. ***p < 0.001, **p < 0.01, *p < 0.05, NS: p > 0.05.

All groups exhibited significant increases in the percentages of MER to the CS with increasing the number of conditioning trials (Fig. 4a, Cochran’s Q test: *OA1*: χ^2^ = 9.8, p = 0.02; *Dop1*: χ^2^ = 32.3, p = 0.00000046; *Dop2*: χ^2^ = 32.2, p = 0.00000048; *DsRed2*: χ^2^ = 31.9, df  = 3, p = 0.00000056). Between-group comparison, however, showed that the percentage of MER to the CS in the *OA1* RNAi group was significantly lower than that in the control (*DsRed2* RNAi) group in the 4th trial (i.e., after the third training, see Fig. 3) (Fisher’s exact test, adjusted by Holm’s method: p = 0.012, sample numbers shown in the figure). In contrast, the percentages of MER to the CS in the *Dop1* and *Dop2* RNAi groups did not significantly differ from that in the *DsRed2* RNAi group (Fisher’s exact test, adjusted by Holm’s method: *Dop1:* p = 0.781, *Dop2:* p = 0.189). The results indicate that silencing of *OA1* significantly reduces acquisition in appetitive learning, but that of *Dop1* or *Dop2* does not. Impairment of appetitive learning by silencing of *OA1* is not due to impairment of the perception of water US, because the crickets exhibited MER to a drop of water applied to the mouth or palpi. Moreover, perception of CS and behavioral responses to the CS are intact in the *OA1* RNAi crickets, as evidenced by their intact aversive learning described below.

Next, retention performance was tested at 30 min after 4-trial appetitive conditioning. The *Dop1*, *Dop2* and *DsRed2* RNAi groups exhibited high percentages (more than 70%) of MER to the odor paired with the US (paired odor or CS), and the percentages were significantly greater than those of MER to the odor not used in training (novel odor) (Fig. 4b, McNemar’s test: *Dop1*: χ^2^ = 11.1, df = 1, p = 0.0000013; *Dop2*: χ^2^ = 9.09, p = 0.0026; *DsRed2*: χ^2^ = 19, p = 0.0000013). In the *OA1* RNAi group, on the other hand, percentage of MER to the CS did not significantly differ from that to the novel odor (χ^2^ = 0, p = 1). Thus, the *Dop1* and *Dop2* RNAi groups exhibited normal retention of CS-specific appetitive memory, but the *OA1* RNAi group did not.

### Effects of the suppression of *OA1, Dop1* and *Dop2* genes on acquisition and retention in aversive conditioning

In aversive conditioning experiment, we used vanilla or maple odor, to which crickets exhibited high percentages (more than 70%) of MER^8^. We observed that repeated presentation of these odors alone led to a decrease of MER percentages^8^, obviously due to habituation. In order to discriminate pairing-specific decrement in percentages of MER due to aversive conditioning from this non-associative effect, we used a differential conditioning procedure in which one of the two odors was paired with 20% sodium chloride solution (paired odor) and the other was presented alone (unpaired control odor) (Fig. 3b)^8^, and the percentages of MER to the paired and unpaired odors were statistically compared.

Four groups of crickets were injected with dsRNA of *OA1*, *Dop1, Dop2* or *DsRed2*, and two days later they received 4-trial differential aversive conditioning training with 5-min inter-trial intervals (Fig. 3). The percentages of MER to the unpaired odor significantly decreased with the increase of the trial number in the *OA1*, *Dop1* and *Dop2* RNAi groups (Cochran’s Q test: *OA1*: χ^2^ = 22.6, p = 0.000050; *Dop1*: χ^2^ = 11.6, p = 0.0088; *Dop2*: χ^2^ = 26.6, p = 0.0000071). *DsRed2* RNAi group also showed a decrease of percentages of MER to the unpaired control odor, but the decrease was not statistically significant (χ^2^ = 3.5, df = 3, p = 0.317). This was obviously due to a slightly lower percentage of MER in the first trial, which appeared to reflect incidental data variation due to small sample size.

**Figure 5:**
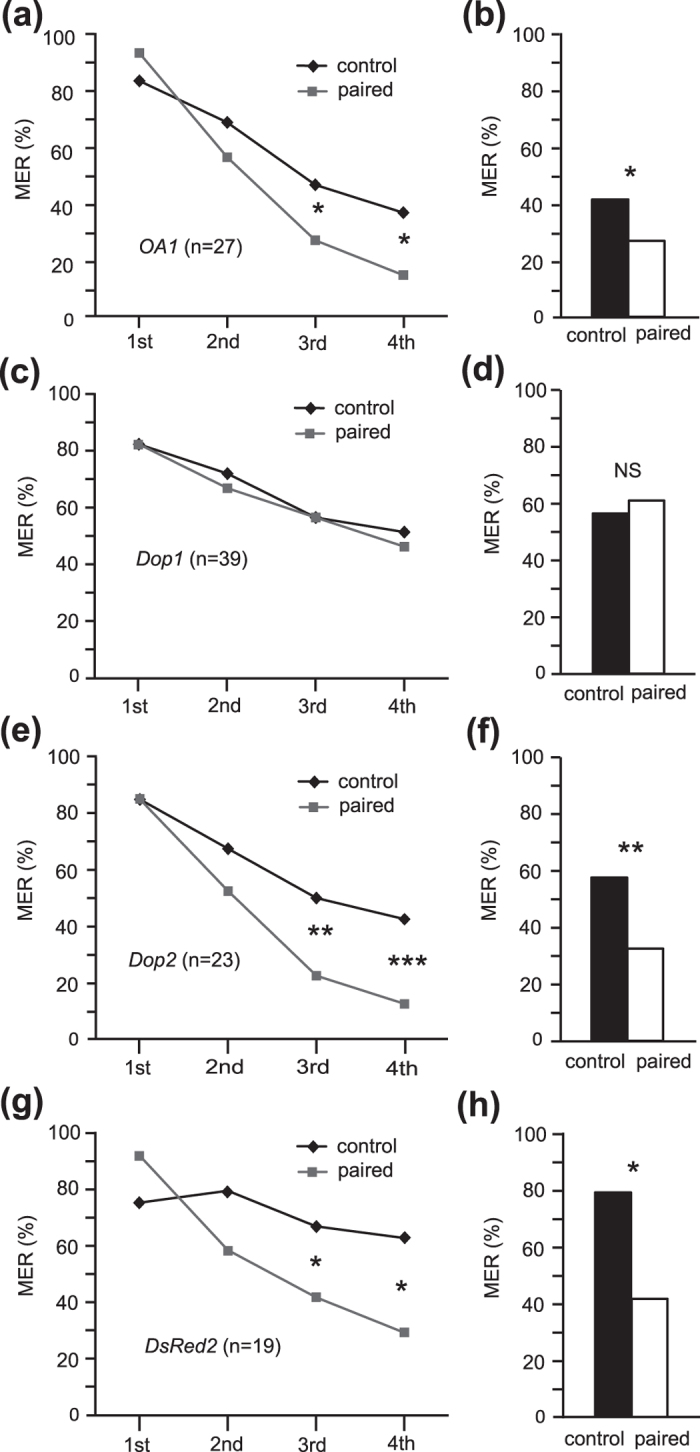
Acquisition and retention in aversive conditioning of *OA1*, *Dop1, Dop2* and *DsRed2* RNAi crickets. (**a,c,e,g**) Acquisition performance of *OA1* (**a**), *Dop1* (**c**)*, Dop2* (**e**) and *DsRed2* (**g**) RNAi groups. Crickets were injected with dsRNA and two days later they were subjected to pairing of an odor with sodium chloride solution as punishment (paired odor) and presentation of another odor alone (unpaired odor) four times each with 5-min inter-trial intervals. Percentages of MERs to the paired odor (gray dots) and those to the unpaired odor (black dots) are plotted. Results of statistical comparison (by Fisher’s exact test) among groups in the 3rd and 4th trials are shown. The sample numbers are shown in parenthesis. (**b,d,f,h**) Retention performance of *OA1* (**b**), *Dop1* (**d**)*, Dop2* (**f**) and *DsRed2* (**h**) RNAi groups at 30 min after conditioning. Percentages of MER to the paired odor (white bar) and unpaired odor (black bar) are plotted. Above the bars, statistical results of McNemar’s test are shown. ***p < 0.001; **p < 0.01; *p < 0.05.

Percentages of MER to the paired odor significantly decreased with progress of training in all groups (Fig. 5, Cochran’s Q test: *OA1*: χ^2^ = 59.9, p = 0.00000000000062; *Dop1;* χ^2^ = 13.7, p = 0.0034; *Dop2*: χ^2^ = 57.5, p = 0.0000000000020; *DsRed2*: χ^2^ = 24.1, df = 3, p = 0.000023). In *OA1*, *Dop2* and *DsRed* RNAi groups, percentages of MER to the paired odor were significantly lower than those to the unpaired odor in the 3rd trial (McNemar’s test: *OA1*: χ^2^ = 4.57, p = 0.033; *Dop2*: χ^2^ = 9.31, p = 0.0023, *DsRed2*: χ^2^ = 4.5, df = 1, p = 0.033) and 4th trial (*OA1*: χ^2^ = 5.4, p = 0.020; *Dop2*: χ^2^ = 12, p = 0.00053; *DsRed2*: χ^2^ = 6.4, p = 0.011), indicating that aversive conditioning is successful. In the *Dop1* RNAi group, on the other hand, the percentages of MER to the paired odor did not significantly differ from those to the unpaired odor in the 3rd trial (McNemar’s test: χ^2^ = 0.29, p = 0.59) and 4th trial (χ^2^ = 0.25, p = 0.62). The results suggest that silencing of *Dop1*, but not that of *OA1* or *Dop2*, impairs acquisition in aversive conditioning.

Retention performance was tested at 30 min after 4-trial differential aversive conditioning. The *OA1*, *Dop2* and *DsRed2* RNAi groups exhibited significantly lower percentages of MER to the CS (paired odor) than to the unpaired odor (Fig. 5, McNemar’s test: *OA1*: χ^2^ = 4.5, p = 0.034; *Dop2*: χ^2^ = 7,14, p = 0.0075; *DsRed2*: χ^2^ = 9, df = 1, p = 0.0027). In the *Dop1* RNAi group, on the other hand, the percentages of MER to the paired odor did not significantly differ from those to the unpaired odor (McNemar’s test, χ^2^ = 0.29, p = 0.59). Thus, the *OA1* and *Dop2* RNAi groups exhibited normal retention of CS-specific aversive memory, but the *Dop1* RNAi group did not.

## Discussion

There has been a discrepancy in the results of studies on neurotransmitters mediating reinforcement signals in crickets and fruit-flies, and the purpose in this study was to fully resolve the discrepancy. Results of studies using transgenic fruit-flies have suggested that different types of dopamine neurons mediate both appetitive and aversive reinforcement signals via the Dop1 receptor^12^,^13^,^14^,^15^,^16^,^34^,^35^. On the other hand, results of our pharmacological studies have suggested that octopamine and dopamine mediate appetitive and aversive reinforcement signals in crickets^3^,^4^,^5^,^6^,^7^,^8^,^36^. In accordance with this, moreover, our recent study using *Dop1*-knockout crickets showed that they are defective in aversive learning but not in appetitive learning^19^. The results of that study, however, were not conclusive, since the aversive learning defects may be due to defects in the development of brain circuitry.

In this study, we observed that *OA1, Dop1* and *Dop2* RNAi crickets exhibited ca. 70–75% reduction in expression levels of these genes. These results suggest that silencing of the expression of these genes is successful, though we could not determine amounts of these receptor proteins in the brains of dsRNA-injected crickets because antibodies against these receptors were not available. We observed that *Dop1*-silenced crickets exhibited impairment of aversive learning but not appetitive learning, whereas *OA1*-silenced crickets exhibited impairment of appetitive learning but not aversive learning. The impairments in RNAi crickets were not due to defects of odor (CS) or water (US) perception or motor function necessary for performance of MER, because the *Dop1*-silenced crickets exhibited normal appetitive MER conditioning and the *OA1*-silenced crickets exhibited normal aversive conditioning. It can be argued that different experimental procedures for appetitive and aversive conditioning (absolute or differential conditioning and the use of different odors) might be the reason for the different effects of *Dop1* and *OA1* gene silencing. However, this is unlikely because we previously showed that effects of dopamine and octopamine receptor antagonists are conserved among experiments with an absolute or differential conditioning procedure and among different kinds of odors used as the CS (see discussion in ref. 8). The consistency between the results of the present gene silencing study with those of the pharmacological and *Dop1* gene knockout study using CRISPR/Cas9 (cited above) strongly suggests that dopamine mediates aversive reinforcement via Dop1 receptors and octopamine mediates appetite reinforcement via OA1 receptors in associative learning in crickets.

Our observation that silencing of *OA1* but not that of *Dop1* or *Dop2* impairs appetitive learning suggests that impairment of appetitive learning by administration of octopamine receptor antagonists observed in our previous studies^3^,^4^,^5^,^6^ is due to their effect on OA1 receptor. We observed that epinastine and mianserin impair appetitive learning but not aversive learning and, since these drugs are known as potent antagonists of insect octopamine receptors^37^,^38^, we suggested that octopamine mediates appetitive reinforcement but not aversive reinforcement. A recent study in honey bees, however, suggested that these drugs antagonize not only OA1 but also Dop2^39^, which raised the possibility that the impairment might be mediated by blockade of the Dop2 receptor, instead of or in addition to the OA1 receptor. The finding in the present study that silencing of *Dop2* does not impair appetitive learning refutes this possibility.

Our suggestion that OA1 but not Dop1 mediates appetitive reinforcement with water reward in crickets fundamentally differs from the suggestion based on results of studies using transgenic fruit-flies that Dop1 mediates appetitive reinforcement with sucrose or water reward in addition to aversive reinforcement^12^,^13^,^14^,^15^,^16^,^34^,^35^. The different conclusions regarding neurotransmitters mediating appetitive reinforcement obtained in studies on crickets and fruit-flies cannot be ascribed to a slight difference in conditioning and testing procedures (see discussion in ref. 8). We thus conclude that neurotransmitters mediating appetitive reinforcement indeed differ in crickets and fruit flies, whereas those for aversive reinforcement are the same.

The results of our studies in crickets raise a question about the diversity and evolution of reinforcement systems for associative learning among animals. There is evidence suggesting that dopamine neurons mediate appetitive reinforcement in mammals^1^,^2^, mollusks^40^ and fruit-flies^14^,^15^, whereas octopamine neurons have been suggested to mediate appetitive reinforcement in honey bees^9^,^10^. The diversity of neurotransmitters mediating appetitive reinforcement among invertebrates and vertebrates should emerge as a fascinating research subject.

The fact that appetitively reinforcing neurons in mammals and crickets use different neurotransmitters may indicate that they mediate different information for reinforcement. Results of our recent study, however, suggested that the signals that these neurons mediate are conserved between mammals and crickets. In mammals, it has been shown that whether appetitive learning occurs is determined by the discrepancy, or error, between the predicted reward and the actual reward^41^ and that certain classes of midbrain dopamine neurons mediate reward prediction errors^1^,^2^. In crickets, our behavioral and pharmacological studies suggest that octopamine neurons mediate reward prediction error^33^. We are currently investigating whether dopamine neurons mediate punishment prediction error in aversive learning in crickets.

We found no impairment in appetitive or aversive learning in *Dop2* RNAi crickets. Since we did not evaluate to what extent the level of Dop2 protein is reduced in Dop2 RNAi crickets and since *Dop2* is known to be expressed in some Kenyon cells in crickets^24^, more studies are needed to examine the possibility that Dop2 plays some roles in olfactory learning and memory. Insects possess several types of octopamine and dopamine receptors other than Dop1, Dop2 and OA1^25^,^26^,^27^. Expression of these genes in the cricket brain and the possible participation of these receptors in learning and memory remain as subjects of our future study.

There is dense expression of *Dop1* mRNA in Kenyon cells of crickets^24^. A high expression level of *OA1* mRNA in Kenyon cells of the mushroom body has been reported in the fruit-fly^31^ and honey bee^32^, and there is an urgent need to confirm this in crickets. The mushroom body is known to play critical roles in olfactory learning in fruit-flies^29^, honey bees^30^, cockroaches^42^ and crickets^43^. Anatomical and physiological characterization of dopaminergic and octopaminergic neurons that make synapses with Kenyon cells is the next step for elucidation of the neural mechanisms of appetitive and aversive learning in crickets.

We conclude that the neurotransmitter and the receptor mediating appetitive reinforcement signals differ in crickets and fruit-flies. This urges us to examine neurotransmitter mechanisms for associative learning in different species of insects to evaluate the diversity, and evolution, of reinforcing mechanisms in insects. Moreover, consideration of the ubiquity and diversity of reinforcing mechanisms for associative learning in vertebrates and invertebrates should become an important research subject in neuroscience.

## Methods

### Insects

Adult male crickets, *Gryllus bimaculatus*, at 1–2 weeks after the imaginal molt were used. They were reared in a 12 h: 12 h light : dark cycle at 27 ± 2 °C and were fed a diet of insect pellets and water *ad libitum*. Four days before the start of the learning trials, crickets were individually placed in 100-ml glass beakers and fed a diet of insect pellets *ad libitum* but deprived of drinking water to enhance their motivation to search for water.

### cDNA cloning of the octopamine receptor gene

We cloned a cDNA fragment containing the partial open reading frame (ORF) of the OA1 octopamine receptor gene by reverse transcriptase polymerase chain reaction (RT-PCR). Total RNA was extracted from the heads of 6^th^ instar nymphs using ISOGEN (Wako, Japan). This allows faster RNA extraction than that from isolated brains since isolation of the brains takes time due to their small size. cDNA was synthesized using the SuperScript III First-Strand Synthesis System (Invitrogen, Carlsbad, CA, UAS). In order to design gene-specific primers to clone a partial cDNA of the *OA1* gene, we searched the cricket genome database (unpublished data) and the following oligonucleotide primers were designed. *OA1* Fw (5′-TTCATGAGCTCCAAGCTGCG-3′) and *OA1* Rv (5′-AAGAGCGCGTAGATGCAGGG-3′). RT-PCR was performed on this cDNA using LA Taq DNA polymerase (Takara, Japan), and the reaction conditions were 94 °C, 2 min for one cycle; 94 °C, 30 s, 55 °C, 30 sec, 72 °C, 1 min for 40 cycles; and then 72 °C, 5 min for one cycle. The PCR product (1544 bp) was subcloned into a pGEM T-Easy vector (Promega, Madison, Wisconsin, USA) and sequenced using ABI-300.

### RNA interference experiments

The procedures for RNAi experiments in this study were according to previous studies^21^,^24^ with a slight modification. Double-stranded RNAs (dsRNAs) of *Dop1* and *Dop2* (*Gb’DopRI* and *Gb’DopRII*)^23^,^24^, *OA1* and *Discosoma sp. red fluorescent protein 2* (*DsRed2*) were synthesized from the corresponding PCR fragments amplified with primers containing the T7 promoter, using the MEGA-script Kit (Applied Biosystems Inc., Foster City, CA). The primer sequences are: *OA1* dsRNA T7-Fw, 5′-TAATACGACTCACTATAGGG AACAAGCCCAAGCTCATCTCG-3′; *OA1* dsRNA T7-Rv, 5′-TAATACGACTCACTATAGGG TTGATGGCGGAGTTGCAGTA-3′; *Dop1* dsRNA T7-Fw, 5′-TAATACGACTCACTATAGGG GGTGTGCGTGGCCATCTA-3′; *Dop1* dsRNA T7-Rv, 5′-TAATACGACTCACTATAGGG TCCTTGATGTGGATGTAGCG-3′; *Dop2* dsRNA T7-Fw, 5′-TAATACGACTCACTATAGGG GTCCTCCTGCTGCTGTTCTC-3′; *Dop2* dsRNA T7-Rv, 5′-TAATACGACTCACTATAGGG ATGACGCACAGGTTGAGGAT-3′.

The dsRNA regions for *OA1*, *Dop1* and *Dop2* genes are shown in Fig. 1. *DsRed2* dsRNA from pDsRed2-N1 (Clontech Laboratories, Inc., Palo Alto, CA) was used as a control. Their target sizes are 233 bp for *OA1*, 252 bp for *Dop1*, 272 bp for *Dop2*, and 660 bp for *DsRed2*. There were some degree of overlap in DNA sequence between *Dop1* and *Dop2* dsRNA regions: The sequence of *Dop1* target region has 71% identity to that of *Dop2*, and that of *Dop2* target region has 69% identity to that of *Dop1* in maximum. However, we observed that aversive learning is impaired in *Dop1* dsRNA-injected crickets but not in *Dop2* dsRNA-injected crickets (see Results section), indicating that cross-reaction, if occurred, is weak.

We performed blast searches using all of the designed dsRNA sequences against the DDBJ database, which includes *G. bimaculatus* EST (cDNAs from early embryos and regenerating nymphal legs, 10,656 sequences) and HTC (high-throughput cDNA sequences from whole bodies of 1st–8th instar nymphs, 32,010 sequences) data, as well as other isolated *Gb* cDNA sequences. Our searches did not identify significant matches of the *OA1*, *Dop1* or *Dop2* dsRNA sequences with any deposited *Gb* sequences other than the target sequences. After the RNA synthesis, the samples were purified, boiled, and gradually cooled down to room temperature for their annealing.

For RNAi experiment, 2 μl of 10 μM dsRNA solution suspended in RNase-free water, was injected into the head hemolymph of each cricket, the method of which is illustrated in Supplementary Figure S1. The cricket injected with dsRNA was individually placed in a beaker for two days until the test of the effect. The amount of injection of dsRNA and the time interval between dsRNA injection and the test were determined based on our previous study^22^.

### Real-time quantitative PCR (qPCR)

Two days after the injection, the heads of crickets were isolated and then homogenized to extract their total RNA using ISOGEN (NIPPON GENE Co., Ltd). The extracted total RNA was reverse-transcribed to cDNA using Superscript-III reverse transcriptase and random hexamers (Invitrogen). For qPCR, primers were designed with Primer Express™ software (Applied Biosystems Inc.). The qPCR was performed using SYBR Green PCR Master Mix (Applied Biosystems Inc.) or THUNDERBIRD SYBR qPCR Mix (ToYoBo Co., Ltd) on a ABI 7300 Real Time PCR System (Applied Biosystems Inc.) under the following conditions: 10 min at 95 °C and then 40 cycles of 15 s at 95 °C, 30 s at 60 °C, and 30 s at 72 °C. *ß-actin* was used as an endogenous reference to calculate the relative expression levels of target genes. *Dop1, Dop2* and *OA1* to *ß-actin* ratios were determined by the comparative Ct method^44^. The qPCR primer sequences are listed below:

*OA1* Fw, 5′-GCCCTTCTTCACCATGTACG-3′;

*OA1* Rv, 5′-GTAGATGCAGGGGTTGATGG-3′;

*Dop1* Fw, 5′-ATCAAGGACCCGCTCAGGTA-3′;

*Dop1* Rv, 5′-CTCCAGCAGCCACACCAC-3′;

*Dop2* Fw, 5′-GAAGAAGGCGGCTAAGACCT-3′;

*Dop2* Rv, 5′-ACAGCCGACACCAATTCTTC-3′;

*β-actin* Fw, 5′-TTGACAATGGATCCGGAATGT-3′;

*β-actin* Rv, 5′-AAAACTGCCCTGGGTGCAT-3′.

### Olfactory conditioning of MER

The procedures for evaluation of olfactory conditioning of maxillary palpi extension response (MER) have been reported elsewhere^8^. Crickets tend to extend their maxillary palpi and vigorously swing them when water is applied to the antennae and when they perceive some but not other odors, which we refer to as MER. High percentages of crickets exhibited MER to vanilla and maple odors but exhibited MER only rarely to apple or peppermint odor, and the former odors were used for appetitive conditioning experiment, while the latter odors were used for the aversive conditioning experiment^8^. Hypodermic syringes (1 ml each) were used to apply CS and US. A small filter paper (3 mm × 3 mm) was attached to the needle of syringe at 10 mm from its tip. The syringe was filled with water (reward) or 20% sodium chloride solution (punishment). The filter paper attached to the needle was soaked with odor essence.

We used an absolute appetitive conditioning procedure for appetitive learning and a differential conditioning procedure for aversive learning, since these are the most effective procedures and since we have shown that neurotransmitters involved in appetitive or aversive conditioning did not differ regardless of the conditioning procedure being absolute or differential^8^. For absolute appetitive conditioning, the filter paper was placed within ~1 cm of the antennae of the cricket for 6 sec and then a drop of water (US) was presented to the mouth for 3 sec (3-sec overlap) four times each with an inter-stimulus interval (ITI) of 5 min. For differential aversive conditioning, a 6-sec CS presentation was similarly followed by 3-sec presentation of sodium chloride solution (US) and another odor was presented alone without pairing with the US four times each with a 5-min ITI. The sequence of the paired trial and unpaired trials was pseudo-randomized. The presence or the absence of MER for the 3-sec period from the onset of CS presentation was recorded.

### Retention test

The procedure for the retention test has been described^8^. For evaluation of retention performance after absolute appetitive conditioning, the percentage of MER to the odor used in training (paired odor) was compared to that to an odor not used in training (novel odor). The odors were presented for 5 sec each with 5-min intervals, and the presence or absence of an MER was recorded. For evaluation of retention after differential aversive conditioning, the percentage of MER to the odor paired with the US (paired odor) was compared to that to the odor presented alone (unpaired odor). After the last odor presentation in the test, a drop of water was attached to the mouth or an antenna and the resulting MER was tested. Crickets that did not exhibit an MER to water US were not used for data evaluation. Crickets discarded by this criterion were less than 3% in total.

### Analysis of behavioral data

We scored the presence or absence of MER to odor presentation in training and in retention tests as reported previously^8^. Data in each group were evaluated by calculating the percentage of MER as the number of crickets that exhibited MER to an odor in the total number of crickets studied. Cochran’s *Q* test was used for comparison of percentages of MER to an odor with the progress of training in acquisition. McNemar’s test was used for pairwise comparison of percentages of MER to two odors in the same group, and Fisher’s exact test was used for pairwise comparison of percentages of MER to an odor in acquisition in different groups. The post-hoc Holm’s method was used to adjust the P value when comparing among more than three groups.

## Additional Information

**How to cite this article**: Awata, H. *et al*. Roles of OA1 octopamine receptor and Dop1 dopamine receptor in mediating appetitive and aversive reinforcement revealed by RNAi studies. *Sci. Rep.* **6**, 29696; doi: 10.1038/srep29696 (2016).

## Supplementary Material

Supplementary Information
