# Supplementary material for: Roles of OA1 octopamine receptor and Dop1 dopamine receptor in mediating appetitive and aversive reinforcement revealed by RNAi studies
Source: Sci Rep. 2016 Jul 14;6:29696. doi: 10.1038/srep29696 (PMC4944188; doi:10.1038/srep29696)
Supplement: Supplementary Information [file srep29696-s1.pdf]

Title: Roles of OA1 octopamine receptor and Dop1 dopamine receptor in mediating appetitive and aversive reinforcement revealed by RNAi studies

Authors: Hiroko Awata, Ryo Wakuda, Yoshiyasu Ishimaru, Yuji Matsuoka, Kanta Terao, Satomi Katata, Yukihisa Matsumoto, Yoshitaka Hamanaka, Sumihare Noji, Taro Mito, Makoto Mizunami

Supplementary Figure S1

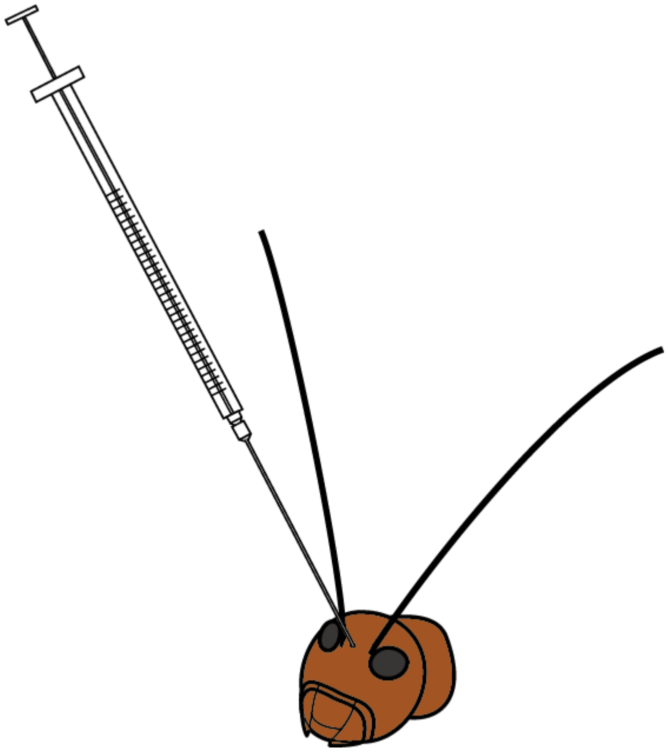

Figure S1. The method of injection of dsRNA solution into the head haemolymph of a cricket. A cricket was removed from a beaker and gently grasped between fingers, and a small hole was made into the head cuticle between the compound eyes using a syringe needle. The cricket was then placed in a beaker until injection. One hour later the cricket was again removed from the beaker, and 2 µl of 10 µM dsRNA solution was injected into the head haemolymph using a 10 µl microsyringe.
